# The Coronary Artery Disease-associated Coding Variant in Zinc Finger C3HC-type Containing 1 (ZC3HC1) Affects Cell Cycle Regulation*

**DOI:** 10.1074/jbc.M116.734020

**Published:** 2016-05-19

**Authors:** Peter D. Jones, Michael A. Kaiser, Maryam Ghaderi Najafabadi, David G. McVey, Allan J. Beveridge, Christine L. Schofield, Nilesh J. Samani, Tom R. Webb

**Affiliations:** From the ‡ Department of Cardiovascular Sciences and; § NIHR Leicester Cardiovascular Biomedical Research Unit, Glenfield Hospital, University of Leicester, Leicester, LE3 9QP and; ¶ Horizon Discovery Limited, 7100 Cambridge Research Park, Waterbeach, Cambridge CB25 9TL, United Kingdom

**Keywords:** cell cycle, cyclin, cyclin-dependent kinase (CDK), phosphorylation, protein structure, single-nucleotide polymorphism (SNP), CAD, coronary artery disease, genome editing, rAAV

## Abstract

Genome-wide association studies have to date identified multiple coronary artery disease (CAD)-associated loci; however, for most of these loci the mechanism by which they affect CAD risk is unclear. The CAD-associated locus 7q32.2 is unusual in that the lead variant, rs11556924, is not in strong linkage disequilibrium with any other variant and introduces a coding change in *ZC3HC1*, which encodes NIPA. In this study, we show that rs11556924 polymorphism is associated with lower regulatory phosphorylation of NIPA in the risk variant, resulting in NIPA with higher activity. Using a genome-editing approach we show that this causes an effective decrease in cyclin-B1 stability in the nucleus, thereby slowing its nuclear accumulation. By perturbing the rate of nuclear cyclin-B1 accumulation, rs11556924 alters the regulation of mitotic progression resulting in an extended mitosis. This study shows that the CAD-associated coding polymorphism in *ZC3HC1* alters the dynamics of cell-cycle regulation by NIPA.

## Introduction

Coronary artery disease (CAD)^3^ is the leading cause of death worldwide. It is caused by a combination of genetic and lifestyle factors that result in progressive development of atherosclerotic plaques, comprising a lipid core and cellular components, in the walls of the coronary arteries (1). The plaques are covered by a fibrous cap of connective tissue and smooth muscle cells, which can become unstable and rupture. This exposes flowing blood to the thrombogenic plaque material causing thrombus formation, which if occlusive can impede blood supply to the downstream myocardium resulting in a myocardial infarction (1).

Genome-wide association studies (GWAS) have identified more than 56 loci, which contribute to risk of CAD (2–7). Many of these loci do not affect known risk factors for CAD, such as blood pressure or LDL-cholesterol levels, which are the primary targets of current treatments for CAD. Therefore, studying the function of associated variants at these loci may identify novel gene pathways contributing to CAD, which may result in the development of novel therapeutics.

The 7q32.2 CAD locus was first reported by Schunkert *et al.* (7). 7q32.2 is unusual, compared with other CAD loci, in that only a single SNP (rs11556924) is associated with CAD risk at this locus, with no other variants in strong linkage disequilibrium. Furthermore, rs11556924 is a coding SNP in the *ZC3HC1* gene, resulting in an arginine-histidine polymorphism at amino acid residue 363 in the NIPA (Nuclear Interaction Partner of ALK) protein encoded by *ZC3HC1* (8). rs11556924 has also been associated with altered carotid intima-media thickness in patients with rheumatoid arthritis (9) and with altered risk of atrial fibrillation (10). The Arg-363 allele is the more common allele (allele frequency = 0.62) (7) and is associated with a 9% increase in CAD risk per allele. This coding change has been predicted to be deleterious to NIPA function (11), but its functional effects have not been investigated.

NIPA is an F-box protein (8). F-box proteins are the targeting subunit of the SCF (Skp1, Cul1, F-box) class of E3 ubiquitin ligases (12, 13). SCF^NIPA^ is only present in the nucleus and acts to ensure degradation of cyclin-B1 during interphase, keeping its levels in the nucleus low (8). Cyclin-B1 is a key regulator of mitotic entry (14); its levels are low during interphase, then it begins to accumulate in the cytoplasm during S-phase, and then ultimately accumulates in the nucleus to promote entry into mitosis (15, 16). The key regulation of Cyclin-B1 occurs by preventing Cyclin-B1 from accumulating in the nucleus where it is required to bind to CDK1 to form the MPF (Maturation-Promoting Factor) complex. Two factors contribute to preventing Cyclin-B1 from accumulating in the nucleus - an atypical nuclear export signal in Cyclin-B1 promotes its export from the nucleus (17, 18) and NIPA acts to degrade any Cyclin-B1 that enters the nucleus therefore preventing its premature accumulation (8). NIPA is therefore an important regulator of mitosis and meiosis (8, 19–21).

**FIGURE 1.**
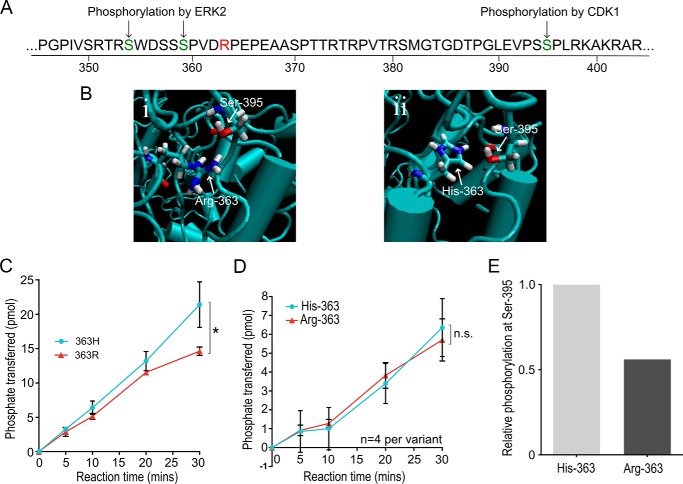
**The CAD associated SNP in ZC3HC1 alters regulatory phosphorylation of the NIPA protein.** *A*, schematic of the secondary structure of NIPA showing the Arg-363-His polymorphism and the regulatory phosphorylation sites at Ser-353, Ser-359, Ser-395. *B*, three-dimensional structure of the NIPA carrying the Arg-363 (*i*) and His-363 (*ii*) variants was predicted. *C* and *D*, radioactive *in vitro* kinase assay time-courses testing phosphorylation of bacterially expressed NIPA carrying each variant. *C*, phosphorylation at Ser-395 by CDK1 kinase (linear mixed model, *p* = 0.003). *D*, phosphorylation at Ser-354 and Ser-359 by ERK2 kinase (linear mixed model, *p* = 0.662). *N* numbers represent independent *in vitro* reactions, carried out across three separate experiments. *E*, mass spectrometric analysis of phosphorylation at Ser-395 (*n* = 1). *Error bars* indicate standard deviation.

The function of NIPA itself is regulated by phosphorylation at key residues, Ser-354 and Ser-359 targeted by the ERK1/2 kinases (20) and Ser-395, which is phosphorylated by CDK1 (19). The Ser-354 and Ser-359 residues lie close to the Arg-363-His residue altered by rs11556924 (Fig. 1*A*). Ser-395 is also in the same region of the protein, although a bit further away in the secondary structure. Given these findings we hypothesized that the Arg-363-His polymorphism in NIPA affects Cyclin-B1 dynamics and cell cycle regulation through an impact on phosphorylation of NIPA and undertook experiments to investigate this. Our study provides a potential functional basis for the effect of allelic variation at rs11556924 on altered CAD risk.

## Results

### 

#### 

##### The rs11556924 SNP Alters Regulatory Phosphorylation of NIPA

To determine if the Arg-363-His polymorphism has the potential to impact on phosphorylation of NIPA, we generated a predicted structural model of the two forms of the protein (Fig. 1*B*). In our model, in the Arg-363 (CAD-associated) variant, the arginine side chain forms a strong hydrogen bond with the Ser-395 residue, the phosphorylation target of CDK1. In the His-363 form of the protein, however, the side chain of the histidine residue points toward the inner core of the leucine rich repeat binding domain and does not interact with Ser-395. There are also conformational changes in the leucine-rich repeat protein binding domain. In particular, the His-363 variant has a looser structure due to the looser winding of several α-helices.

To experimentally confirm whether these changes in the structure of the protein are sufficient to alter the phosphorylation of the protein in this region, we used *in vitro* kinase assays. To achieve this, the 2 NIPA variants, tagged with MBP (maltose-binding protein), were bacterially expressed and then used as substrates for a kinase assay using recombinant CDK1 and ERK2 kinases. MBP alone was used as a negative control and was not phosphorylated. A kinase assay using CDK1 kinase showed that phosphorylation of NIPA occurred at a mean rate of 0.494 ± 0.044 pmol phosphate/min in the Arg-363 variant compared with 0.694 ± 0.141 pmol phosphate/min in the His-363 variant, so phosphorylation is occurring significantly slower in the CAD-risk variant of the protein (*p* = 0.002) (Fig. 1*C*). Phosphorylation by ERK2, however, showed no significant difference between phosphorylation of the 2 NIPA variants (*p* = 0.622), with a mean rate of 0.184 ± 0.065 pmol phosphate/min in the His-363 variant and 0.198 ± 0.025 pmol phosphate/min in the Arg-363 (Fig. 1*D*).

To further validate this effect of rs11556924 on the regulatory phosphorylation of NIPA, we used MS/MS proteomics to carry out phospho-analysis of the NIPA protein. To achieve this, vectors expressing each variant were generated and expressed in HEK293T cells. We then immunoprecipitated NIPA and used MS/MS to examine its phosphorylation. We enriched for phospho-peptides using TiO_2_ beads and used SILAC quantitation to look for differences in the level of phosphorylation at these sites. A total of 39 phosphorylation sites in NIPA were identified in our phospho-enriched samples. We were able to quantify the relative level of phosphorylation at 30 of these sites. Phosphorylation at Ser-395 was measurable using this approach, but phosphorylation at Ser-354 and Ser-359 was not, due to low levels of peptide detection. The site at Ser-395 had 44% lower phosphorylation of the Arg-363 variant compared with the His-363 variant (Fig. 1*E*), suggesting that the polymorphism is perturbing phosphorylation at this site.

##### Generation of Isogenic Lines Differing Only at rs11556924 using rAAV Genome Editing

**FIGURE 2.**
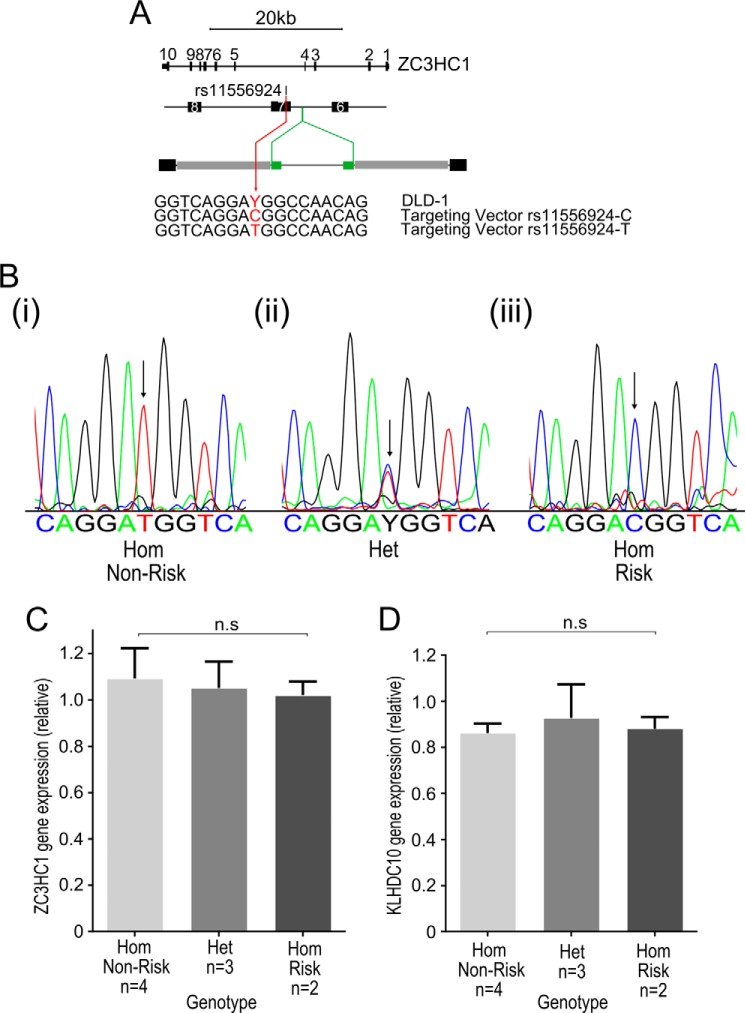
**Genome editing of the rs11556924 CAD-associated SNP.** *A*, rAAV genome editing was used to generate isogenic cell lines. The parental cell line used was DLD-1, which is heterozygous for the SNP. We targeted DLD-1 with rAAV carrying either the C (Risk) or T (Non-Risk) allele to generate isogenic cell lines carrying all three genotypes. *B*, lines were confirmed by sequencing across the region containing the SNP; example sequence traces of (*i*) homozygote non-risk, (*ii*) heterozygote, and (*iii*) homozygote risk genotypes are shown. *Arrows* mark the site of the rs11556924 SNP. *C* and *D*, qRT-PCR was used to compare expression of (*C*) ZC3HC1 (*p* = 0.442) and (*D*) KLHDC10 (*p* = 0.291) between the genome edited cell lines of different genotypes. *N* numbers represent individual cell lines, reactions were carried out in technical triplicates, and data combined from two independent experiments. *Error bars* indicate standard deviation.

Having shown that the coding variation caused by rs11556924 alters regulatory phosphorylation of NIPA, we next investigated the effect of the SNP on endogenous protein function in cells. To do this we created isogenic lines differing only at rs11556924, using a recombinant Adeno-Associated Virus (rAAV) genome editing approach (Fig. 2*A*). rAAV is an effective method for generating small genetic alterations (22, 23). rAAV genome editing relies on homologous recombination for targeting and does not generate double-strand breaks or cause the off-target effects, which have been associated with nuclease-based methods of genome editing (31). As the role of *ZC3HC1* in CAD is unknown, it is uncertain which cardiovascular cell type is most relevant. Also, it is not possible to generate clonal knock-in cell lines in primary cell types. For these reasons, we carried out genome editing in the pseudo-diploid colon carcinoma cell line DLD-1, which has been extensively used as a target cell line for this type of genome editing (22, 24–27). The DLD-1 cell line, which is heterozygous for the SNP, was targeted with rAAV carrying each allele of rs11556924 allowing us to knock in either genotype, generating 4 homozygote CAD-non-risk lines, 4 heterozygote lines (with a recombination event but no change in genotype) and 3 homozygote CAD-risk lines. Genotypes were confirmed by sequencing across the SNP (Fig. 2*B*). Examining multiple lines of each genotype helps to ensure that differences between lines of different genotypes are down to the changes generated at rs11556924 and not due to off-target effects.

A previous study had suggested that rs11556924 may be associated with expression of the *KLHDC10* gene, which is the next gene downstream from *ZC3HC1* (19kb away) (28). To test for an effect on the expression of *KLHDC10*, and also to test whether rs11556924 had any effect on expression of *ZC3HC1* itself, we examined mRNA levels of both genes in the genome edited lines of all 3 genotypes using qRT-PCR. There was no change in expression of either *ZC3HC1* (*p* = 0.442) (Fig. 2*C*) or *KLHDC10* (*p* = 0.291) (Fig. 2*D*), indicating that the SNP does not alter expression of either of these genes.

##### The rs11556924 SNP Alters Regulation of Cyclin-B1 Levels by NIPA

Phosphorylation of NIPA at Ser-395 acts to deactivate NIPA, preventing it from ubiquitinating Cyclin-B1 and thus allowing accumulation of Cyclin-B1 in the nucleus. We therefore next studied the rs11556924 genome edited lines to test whether the SNP genotype impacted on Cyclin-B1 stability.

**FIGURE 3.**
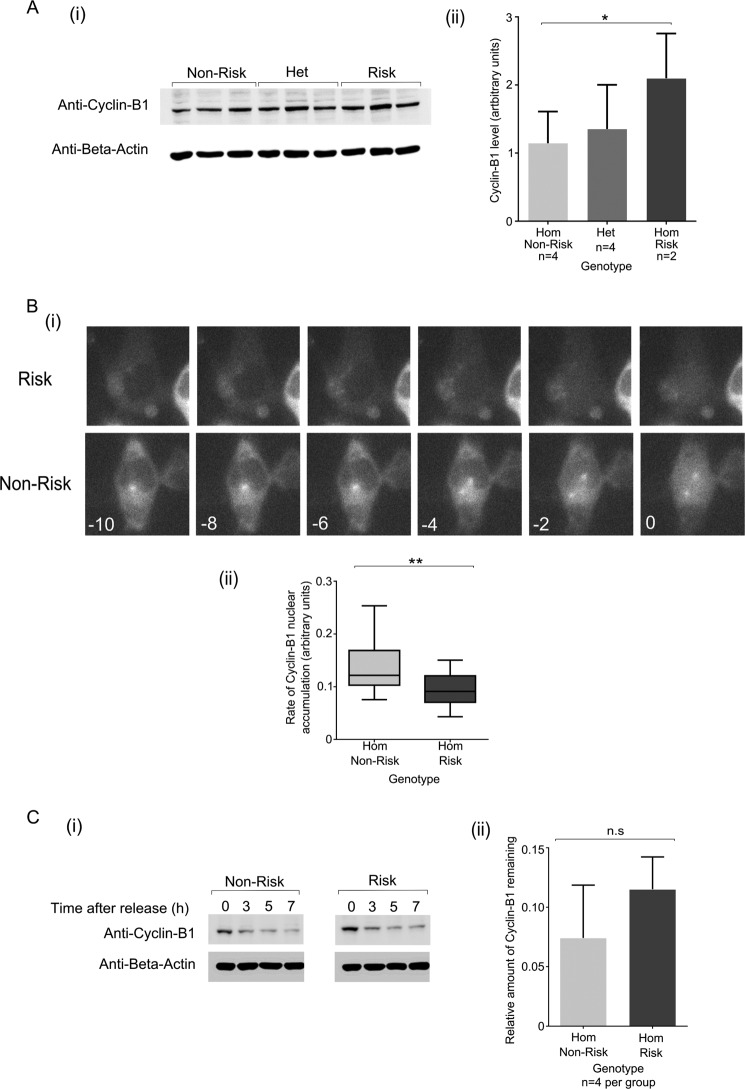
**The rs11556924 SNP alters cyclin-B1 dynamics.** *A*, level of Cyclin-B1 in isogenic cell lines of different genotypes was examined using quantitation of Western blots. Example blot shown in (*i*), quantitation relative to β-actin expression shown in (*ii*) (*p* = 0.017). *N* numbers represent individual cell lines, reactions were carried out in technical triplicates, and data combined from two independent experiments. *B*, to examine the rate of cyclin-B1 nuclear accumulation, Cyclin-B1-GFP was expressed in CAD-risk and CAD-non-risk cells and its live cell imaging used to monitor its accumulation at 2-min intervals. (*i*) In these examples, in the risk cells, nuclear import begins at −6 min, in the risk example, nuclear accumulation begins at −4 min (0 min is the time at which the nuclear/cytoplasmic ratio rises above 1). (*ii*) Boxplot showing overall data for CAD-risk and CAD-non-risk genotype cells (*p* = 0.004). *C*, to further examine the stability of Cyclin-B1 in our isogenic cell lines, we blocked protein synthesis with cycloheximide, and then examined the level of Cylin-B1 after 5 h of treatment. Example blot shown in (*i*) from these data, the relative amount of Cyclin-B1 remaining after 5 h shown in (*ii*) (*p* = 0.167). Data were combined from four experiments of 2 cell lines of each genotype. *Error bars* indicate standard deviation.

We first examined total Cyclin-B1 in cells of the three genotypes using Western blotting with an α-Cyclin-B1 antibody. We found that Cyclin-B1 levels were higher in CAD-risk genotype cells compared with CAD-non-risk cells, with the heterozygote intermediate between the homozygotes (Fig. 3*A*). The overall trend was significant (ANOVA *p* = 0.017) and post-hoc tests showed a significant difference between homozygotes (*p* = 0.015). This finding was contrary to our expectations, a reduction of NIPA phosphorylation in the CAD risk genotype group would have been expected to result in more active NIPA and consequently lower levels of Cyclin-B1. To further examine the impact of rs11556924 on Cyclin-B1 regulation we needed to examine the impact of rs11556924 on its stability in the nucleus more directly.

To investigate the effect of rs11556924 on the nuclear stability of Cyclin-B1, we examined the effect of genotype on Cyclin-B1 at the time in the cell cycle when this effect is likely to be most important, the time of mitotic entry as cyclin-B1 begins to accumulate in the nucleus. We hypothesized that the rate of nuclear accumulation could be altered, perturbing cell cycle dynamics. To test this hypothesis, we expressed Cyclin-B1-GFP in the genome edited cell lines, and used live microscopy to monitor the nuclear entry of Cyclin-B1-GFP (Fig. 3*B*, *i*). We then quantified the relative amount of Cyclin-B1-GFP in the nucleus at 2 min intervals in the time around mitotic entry but prior to nuclear-envelope breakdown. The rate of nuclear entry of Cyclin-B1-GFP was reduced in cells of the risk genotype with risk cells having a mean rate of 0.095 ± 0.032 compared with 0.134 ± 0.048 in cells of the non-risk genotype (*p* = 0.004) (Fig. 3*B*, *ii*).

To confirm that the effect of rs11556924 on Cyclin-B1 nuclear accumulation was due to altered stability of Cyclin-B1, we tested the effect of rs11556924 on the stability of endogenous Cyclin-B1. To achieve this, we blocked protein synthesis in our genome edited cell lines using cycloheximide. Total Cyclin-B1 levels were determined by Western blotting analysis in cells treated with cycloheximide for 5 h and then the relative amount of Cyclin-B1 quantified (Fig. 3*C*). There was a trend toward a lower amount remaining in the CAD-risk genotype cells compared with CAD-non-risk, although this difference was not significant (*p* = 0.167).

##### Alteration of Cyclin-B1 Regulation by rs11556924 Alters Mitotic Progression

**FIGURE 4.**
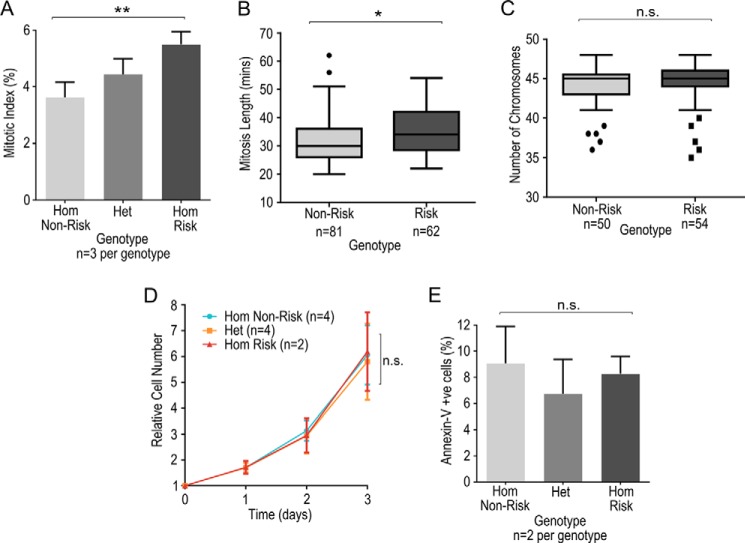
**Phenotypic assessment of the effect of rs11556924 on mitotic progression.** *A*, mitotic index in isogenic cells carrying different genotypes at rs11556924 was determined by fixing cells and staining with DAPI and anti-phosho-histone H3 (*p* = 0.012). Data were combined from three experiments of 2 cell lines of each genotype. *B*, effect of rs11556924 genotype on length of mitosis as assessed by live cell imaging (*p* = 0.011). *N* represents number of mitoses measured across 2 independent cell lines of each genotype from 3 experiments. *C*, chromosome counts in isogenic cells with risk and non-risk rs11556924 genotype (*p* = 0.6516). *N* represents number of cells analyzed, across two independent cell lines of each genotype from three experiments. *D*, cellular proliferation rates in cells of the different rs11556924 genotypes (mixed model on log2 data, *p* = 0.522). *E*, proportion of apoptotic cells detected by flow cytometry using an anti-annexin-V antibody coupled to FITC in isogenic cells of different rs1156924 genotypes (*p* = 0.251). *N* numbers represent individual cell lines, reactions were carried out in technical triplicates, and data combined from two independent experiments. *Error bars* indicate standard deviation.

Having demonstrated that rs11556924 alters the ability of NIPA to regulate the levels of Cyclin-B1 in the nucleus, we next investigated the effect of this altered regulation on control of the cell cycle. Initially, we tested the effect of rs11556924 genotype in the genome-edited cell lines on the mitotic index of these cells, *i.e.* the proportion of cells in mitosis at any one time. We found an increase in the mitotic index of the cells of the CAD-risk genotype, with a mean of 5.50 ± 0.26% compared with CAD-non-risk cells with a mean of 3.62 ± 0.30% with the heterozygote again showing an intermediate phenotype (Fig. 4*A*) (ANOVA *p* = 0.012). Post-hoc testing showed a significant difference between homozygotes (*p* = 0.010).

There are several possible explanations for this higher mitotic index in cells with the CAD risk genotype, the CAD-risk cells could be proceeding through the cell cycle more quickly, which would result in faster cellular proliferation. Alternatively they could be arresting in mitosis due to perturbation of Cyclin-B1 dynamics leading to mitotic errors, or simply taking longer to proceed through mitosis, which could result in slower proliferation.

Given our observation that cells of the risk genotype accumulate Cyclin-B1 into the nucleus more slowly (Fig. 3), our hypothesis was that the most likely explanation for the higher mitotic index in these cells was that they were taking longer to complete mitosis. To test this we used live imaging of cells of the non-risk and risk homozygote genotypes and measured the time taken to complete mitosis. There was an increase in the mean time taken to complete mitosis in the cells carrying the risk genotype, with a mean of 35.4 ± 1.1 min compared with a mean of 32.0 ± 0.9 min in non-risk genotype cells (*p* = 0.011) (Fig. 4*B*).

To test whether perturbation of Cyclin-B1 dynamics resulted in errors in mitosis, which could in turn result in a longer mitosis, we assessed the success of chromosome segregation by carrying out chromosome spreads of metaphase cells and counting the number of chromosomes in each cell. Errors in chromosome segregation would be expected to cause a widening in the distribution of chromosome number in the cells. We did not see this effect; the variation in chromosome number was the same in cells of the non-risk and risk genotypes (*p* = 0.652) (Fig. 4*C*).

##### rs11556924 Genotype and Cell Proliferation

As the dynamics of Cyclin-B1 accumulation and the duration of mitosis are perturbed by rs11556924, this would be expected to alter the rate of proliferation of the cells. In order to test the effect of the SNP on proliferation, we used a Sulforhodamine-B assay of cell number across 72 h of cell growth in the genome-edited lines. There was no difference between cells of the different genotypes (*p* = 0.522) (Fig. 4*D*).

Given that in the CAD-risk genotype cells, mitosis is taking longer, but these cells do not proliferate more slowly, we wondered whether this apparent contradiction was caused by decreased apoptosis in the CAD-risk cells. To test this, we measured apoptosis in the cell lines using a flow cytometry annexin-V assay. We found no detectable difference in the levels of apoptosis between cells of different rs11556924 genotypes (*p* = 0.251) (Fig. 4*E*).

## Discussion

GWAS studies have been very fruitful in identifying SNPs that are associated with altered risk of various complex diseases, including CAD. In many cases, there are several variants in high linkage disequilibrium at each locus that are associated with disease and identifying the causal SNP can be challenging (11). As a result, it is often difficult to predict which gene or genes are the causal ones at a particular CAD-associated locus. Even where the causal gene at a locus is easily predicted, many of those genes do not have known roles in cardiovascular function. Functional analysis of these genes and variants may increase our understanding of the processes of CAD and therefore have the potential to lead to novel therapeutic targets.

The rs11556924 SNP in the *ZC3HC1* gene has been shown to be associated with CAD, with a 9% increased risk with each additional copy of the CAD-associated allele (7). In this case, the SNP is a coding change in the gene, resulting in an amino acid substitution from arginine to histidine. The effect of this substitution on protein function is as yet unknown.

In this study, in addition to investigating the effect of the rs11556924 polymorphism on the biochemistry of the NIPA protein encoded by the *ZC3HC1* gene, we have utilized a genome editing approach to investigate the effect of the SNP on *ZC3HC1* gene function in human cells. This is a powerful approach for investigating the function of SNPs identified from GWAS studies (29), as the effects of these SNPs on gene function are likely to be subtle. This is particularly the case for coding SNPs, where any substantial alteration in protein structure or function would be expected to be largely deleterious. Genome editing as used here allows the study of the effects of the SNP alone in an isogenic background. This approach prevents differences in genetic background from masking what may be a subtle effect of the alteration of a single SNP (30, 39). Generating multiple lines of each genotype ensures that any differences seen are due to the SNP itself and not due to either genetic drift or off-target integration of the rAAV, although off-target events are very rare (22).

We demonstrate that the rs11556924 arginine variant that is associated with CAD risk perturbs the function of NIPA, resulting in an alteration in Cyclin-B1 dynamics. Specifically, the CAD risk variant alters the rate of regulatory phosphorylation of NIPA by Cyclin-B1-CDK1, decreasing the level of NIPA phosphorylation in cells of the risk genotype (Fig. 1*C*). Consequently, NIPA activity is prolonged in cells carrying the CAD-risk genotype, causing slower accumulation of Cyclin-B1 in the nucleus of these cells (Fig. 3). This results in an increase in the time taken to complete mitosis presumably because Cyclin-B1 has to reach a certain threshold level to initiate chromosome segregation (32, 33). Although there is an apparent disparity between lowered phosphorylation of the CAD-risk variant NIPA (Fig. 1*C*) and an increase in total Cyclin-B1 (Fig. 3*A*), this is explained by the slowed progression through mitosis of cells of the CAD risk genotype, resulting in an increased mitotic index (Fig. 4*A*). As there are more cells in mitosis, the average level of Cyclin-B1 in the cells appears higher, although in fact it accumulates more slowly in the nucleus (Fig. 3*B*).

The cell cycle is known to be important in the development of cardiovascular disease (34). This includes its role in the determination of vascular wall structure during development which could subsequently make the vascular wall more or less to prone to damage and development of atherosclerosis. Also important is perturbation of the cell cycle during the proliferative response to vascular injury, especially of smooth muscle cells and the endothelium, which plays a critical role in the development of the atherosclerotic plaque (35, 36). In addition, perturbation of proliferation of specific cell types can alter the composition and strength of the fibrous cap which determines the risk of plaque rupture (37). It has also been shown that Cyclin-B1 and its regulator NF-Y are important in the regulation of plaque growth (38) and neointima formation (39). It is also known that vascular smooth muscle cells in the vessel wall have low turnover, but during atherogenesis and upon in response to vascular injury, cell proliferation substantially increases (36). The effect of the rs11556924 polymorphism in *ZC3HC1* on Cyclin B1 dynamics and the cell cycle could therefore influence the development and progression of CAD through effects on multiple cell types and at different stages of the chronic process of CAD. It should be emphasized that the DLD-1 cell line was used in these studies. Although the effect of the rs11556924 on NIPA function is likely to be generic, further investigation will be required to confirm these effects in CAD relevant cell types.

In the DLD-1 cells used in our study, the impact of rs11556924 on Cyclin-B1 dynamics and mitosis did not result in discernible differences in the rate of cellular proliferation between CAD-risk genotype and CAD-non-risk genotype cells. Our additional analysis suggests that this effect is not due to increased apoptosis in the non-risk genotype cells. There are several possible explanations for this. It is possible that under basal conditions the cells are able to compensate for this delay at other stages of the cell cycle and additional stimuli or stresses may be required to bring out the impact. It is most likely, however, that any difference in proliferation would be subtler than we are able to detect. Given the small difference between mitosis length in the non-risk and risk genotype cells (difference between means of 3.4 min, Fig. 4*B*), considering that the entire cell cycle length is 27.7 h (calculated from proliferation assay data in Fig. 4*D*), it is not surprising that we did not observe a difference in proliferation. A difference of 3.4 min in a cell cycle of 27.7 h would correspond to a difference of 0.2% in the total cycle length. Our proliferation assay was across ∼3 cell cycles, so you would expect approximately a 0.6% in cell number at the end of the assay. Clearly, such a small difference would be extremely challenging to detect in a proliferation assay. Of course, in the context of coronary artery disease, even a subtle difference in cell cycle regulation could have a substantial impact on disease progression over the course of the months and years over which CAD develops.

In summary, we show that the CAD-associated SNP rs11556924 in the ZC3HC1 gene affects phosphorylation of its cognate protein NIPA, thereby altering its ability to influence Cyclin-B1 accumulation in the nucleus and impacting on the progression of the cell through mitosis. These findings provide a better understanding of the functional impact of rs11556924.

## Experimental Procedures

### 

#### 

##### In Silico Protein Structure Prediction

The I-Tasser structure prediction program (40) was used to generate a model for NIPA, based on protein threading. The initial sequence alignment was generated using the transport inhibitor response 1 (TIR1) protein (3C6P) (41), which contains an F-box domain and a leucine-rich repeat (LRR). This model was then refined using a combination of molecular mechanics (MM) and molecular dynamics (MD). The initial structure was optimized (in the gas-phase, 1000 cycles MM energy minimization) using the MD program, NAMD (23) and the CHARMM force field (42, 43). Counter ions (Na^+^) were then added using the program, Ionize, to ensure that the system was electrically neutral. This structure was then solvated in a box of water. The dimensions of the box were chosen to ensure the protein was surrounded by a boundary of at least 8 Å of water in all directions. The solvated system was then subjected to 50,000 cycles of MM energy minimization, before performing a 1-ns MD simulation. Starting from the structure generated from the previous MD simulation of the fully solvated wild-type, Arg-363 was mutated to His^+^-363. The mutant system was then energy minimized (MM) for 50,000 cycles before performing a 1 ns MD simulation.

##### Generation of Vectors Containing Both Variants of ZC3HC1

The wild-type *ZC3HC1* open reading frame (ORF) with a C-terminal FLAG tag in a pCMV-Entry vector was obtained from Origene (Rockville). A QuikChange site-directed mutagenesis kit (Agilent, Santa Clara, CA) was then used to mutate the wild-type (CAD-risk) version to the mutant (CAD-non-risk) version of the gene. The two versions of the ORF were then subcloned into the pLEICS-10 vector, which contains an N-terminal MBP directly prior to the ORF, by The Protein Expression Laboratory at the University of Leicester (Leicester, UK).

##### In Vitro Analysis of NIPA Phosphorylation

The vectors expressing MBP-tagged versions of *ZC3HC1* were transformed into BL21 (DE3) competent cells. To express the protein, bacteria were culture overnight, then a low-density culture seeded; this was allowed to grow for 2 h, then expression induced by the addition of 1 mm IPTG to the culture. Cells were allowed to express for 4 h, then pelleted by centrifugation, the pellets frozen, then protein extracted in PBST. Amylose resin (NEB, Hitchin, UK) was used to purify the proteins according to the manufacturer's instructions. Purified proteins were used as a substrate in a kinase reaction using a commercial kinase buffer (NEB, Hitchin, UK), 200 μm ATP, 2 μCi ATP^32^ and 1 unit of kinase. The reactions were stopped using phosphoric acid, followed by liquid scintillation counting using Ultima Gold scintillation fluid (Perkin-Elmer, London, UK) and a Tri-Carb scintillation counter (Perkin-Elmer).

##### Mass Spectrometric Analysis of NIPA Protein Phosphorylation

The FLAG-tagged proteins were expressed in HEK293T cells grown with SILAC amino acids, “Light” samples contained standard l-arginine-HCl and l-Lysine-HCl, “Heavy” samples contained l-arginine-HCl (U-13C6) and l-lysine-2HCl (4,4,5,5-D4) (CKGas, Leicester, UK). Dialyzed fetal bovine serum (Sigma-Aldrich) was used to avoid amino acid contamination from the serum. Cells were lysed, and anti-FLAG M2 beads (Sigma-Aldrich) used to immunoprecipitate the protein using standard methods. Immunoprecipitated proteins were separated by SDS-PAGE, and the band corresponding to NIPA was excised. The band was then trypsinized, phospho-peptides purified using TiO_2_ beads, the unbound (non-phospho peptides) were kept and analyzed separately and subjected to MS/MS on an LTQ Orbitrap by the University of Leicester Proteomics Facility PNACL. Data were analyzed using MaxQuant (ver. 1.5.0.30) (44). N-terminal acetylation and oxidation (M) were set as variable modifications; carbidomethylation (C) was set as a fixed modification. The appropriate software settings were used to detect the relevant light and heavy SILAC labels for relative protein quantification.

##### Generation of Isogenic Cell Lines by Genome Editing

Recombinant AAV targeting vectors were produced for us by Horizon Discovery (Cambridge, UK). rAAV viruses were generated following transfection of the appropriate targeting and helper vectors in HEK293T cells, and rAAV viruses purified using an AAV purification kit (Virapur, San Diego, CA). AAV was titrated using qPCR. Wild-type DLD-1 cells were infected with the rAAV viruses carrying both alleles of rs11556924 to generate cells of all three genotypes. Following infection cells were selected for using the G418 resistance cassette included in the vector, and selected clonal populations expanded and genotyped by PCR and sequencing (Fig. 2*B*). The loxP-flanked selection cassette was then removed using *Cre* recombinase to leave a single loxP site, the location of which was designed to be unlikely to be detrimental. Clones were then expanded from single cells, the removal of the selection cassette confirmed by PCR, then were genotyped again to ensure correct isolation of clonal lines. This resulted in the generation of cell lines that were isogenic other than at rs11556924.

As the loci that are identified from GWAS have additive effects on CAD risk, it would be expected that any differing phenotypes between risk and non-risk genotypes would be intermediate in the heterozygote, consistent with a dosage effect. Therefore, it is important to obtain heterozygote knock-in lines as well as the homozygotes, to be able to test this.

##### Gene Expression Analysis

RNA was extracted using an RNeasy miniprep kit (Sigma-Aldrich), reverse transcribed with Superscript III (Fisher Scientific), and qPCR carried out with SensiMix (Bioline, London, UK) on a Rotorgene Q qPCR machine (Qiagen, Manchester, UK).

##### Determination of Cellular Cyclin-B1 Levels

Cellular Cyclin-B1 levels were measured using Western blotting analysis, carried out using standard procedures on total cell lysates with an anti-Cyclin-B1 antibody raised in mouse (1:1000) (Santa Cruz Biotechnology) and an anti-mouse IgG from sheep (1:30,000) (Fisher Scientific).

##### Live Cell Imaging

A Nikon Eclipse Ti microscope equipped with an LED light source for epifluorescence and a Nikon Perfect Focus System was used. The microscope has an environmental chamber with temperature control and CO_2_ supply. An Andor iXonEM+ EMCCD DU 885 camera is attached to the microscope for image collection using NIS-Elements software (Nikon Instruments Europe, Amsterdam, The Netherlands). Mitosis measurements were on untreated cells. For Cyclin-B1-GFP imaging, cells were transfected with a vector expressing GFP-Cyclin-B1 (pCMX/cyclinB1-GFP (45) was a gift from Jonathon Pines (Addgene plasmid 26061)) using Lipofectamine LTX (Fisher Scientific). Images were analyzed using the FIJI release of ImageJ (ver. 1.50a) (46).

##### Cellular Assays

For proliferation assays, cells were plated in a 96-well plate, 1000 cells/well, with 8 replicates of each sample. The sulforhodamine-B proliferation assay was used.

Mitotic index was measured by fixation of cells and staining with DAPI (Fisher Scientific) and anti-phospho-Histone-H3 (NEB, Hitchin, UK).

Chromosome counts were performed by trypsinizing the cells, treating them with a hypotonic solution, and then spreading them onto a microscope slide. The DNA was then stained with DAPI, and imaged using an EVOS Fluorescent Microscope (Fisher Scientific). Images were analyzed using the FIJI release of ImageJ (ver. 1.50a) (46). Apoptosis was measured using an Annexin-V-FITC antibody (Biolegend UK) and a CYAN ADP flow cytometer using standard methods.

##### Statistics

Statistical analysis was carried out using R (47) and GraphPad Prism (GraphPad Software). Kinase assay data were analyzed by generating a linear mixed model using the NLME package in R (R Core Team, 2013). Proliferation assay data were log2 transformed and an NLME mixed model used to test significance. For apoptosis assays, raw flow cytometry apoptosis data were analyzed by a custom script using R and the flowCore and flowStats Bioconductor packages (48) and significance determined by ANOVA (47, 49). For gene expression, Cyclin-B1 level and mitotic index data, an ANOVA was used to test for differences between genotypes, post-hoc test used Tukey's test. For Cyclin-B1 nuclear stability and degradation experiments, a Student's *t* test was used to test for differences between genotypes. For chromosome number, an F-test was used to compare variances. All barcharts represent mean ± S.D. Significance levels: n.s., *p* > 0.05; *, *p* ≤ 0.05; **, *p* ≤ 0.01.

## Author Contributions

P. D. J., M. A. K., N. J. S., and T. R. W. designed the experiments. P. D. J., M. A. K., M. G. N., and M. G. M. carried out experiments and analyzed data. A. J. B. carried out protein modelling. C. L. S. designed and generated rAAV vectors. P. D. J., N. J. S., and T. R. W. wrote the paper. All authors analyzed the results and approved the final version of the manuscript.

## Footnotes

3 The abbreviations used are:

CAD: coronary artery disease
MD: molecular dynamics
CDK: cyclin-dependent kinase
SNP: single nucleotide polymorphism
NIPA: nuclear interaction partner of ALK
ORF: open reading frame.
